# Draft Genome Sequence of *Rhizobium* sp. H41, a Rock-Weathering Bacterium from a Weathered Rock Surface

**DOI:** 10.1128/genomeA.01127-14

**Published:** 2014-11-06

**Authors:** Jun Xi, Xiafang Sheng, Linyan He

**Affiliations:** Key Laboratory of Agricultural and Environmental Microbiology, Ministry of Agriculture, College of Life Science, Nanjing Agricultural University, Nanjing, People’s Republic of China

## Abstract

*Rhizobium* sp. H41 isolated from weathered tuff can weather tuff and release Fe, Si, and Al from the rock under nutrient-poor conditions. Here, we report the draft genome sequence of strain H41, which may facilitate a better understanding of the molecular mechanism involved in rock weathering by the bacterium.

## GENOME ANNOUNCEMENT

Rock or mineral weathering is one of the most important geochemical phenomena. Microbial communities, as free-living cells or in biofilms, are the first colonizers of newly exposed rocks (1). They may influence the rate of mineral weathering and the amount and the quality of organic matter in soil (2). Strain H41 was isolated from a weathered tuff surface using the dilution plating method. A rock-weathering experiment showed that strain H41 was able to weather tuff and release significantly more Si, Al, and Fe from the rock than the control. In order to further understand the molecular mechanism of rock weathering by *Rhizobium* sp. H41, we sequenced the whole genome of *Rhizobium* sp. H41.

Genomic DNA of *Rhizobium* sp. H41 was extracted and purified following the methods listed in Wilson’s study (3). The quality of DNA was examined using a NanoDrop2000 spectrophotometer (Thermo Scientiﬁc). The genome of strain H41 was sequenced using Solexa paired-end sequencing technology (4) by Shanghai Majorbio Bio-pharm Technology Co., Ltd. (Shanghai, China). A library with a fragment length of 300 bp was constructed, and a total of 1,929,531 paired-end reads were generated, resulting in 157-fold depth of coverage. The resulting product was cleaned by removing the adapter, low-quality reads, poly-N, and error pair-end reads. The whole genome was assembled and rearranged using SOAPdenovo version 1.05 (http://soap.genomics.org.cn). The open reading frames (ORFs) and rRNA and tRNA genes annotation were performed using the Prokaryotic Genomes Automatic Annotation Pipeline (PGAAP) provided by the National Center for Biotechnology Information (NCBI) (5). Functional classiﬁcation was performed by aligning the predicted proteins to the Clusters of Orthologous Groups (COG) database (6).

The draft genome sequence of *Rhizobium* sp. H41 contains 5,570,737 bp with an average G+C content of 59.33%. All generated reads were assembled into 167 contigs (*N*_50_, 198,490 bp) and rearranged in order into 147 scaffolds. The genome sequence includes 5,294 candidate protein-coding genes, giving a coding intensity of 85%, and the average size of each gene is 894 bp. The genome was shown to encode at least 10 predicted RNAs, including 2 rRNAs and 8 tRNAs, and is involved in 176 predicted metabolic pathways.

According to the annotation results, many genes and metabolic pathways (signal transduction mechanisms, amino acid transport, and metabolism) may be involved in rock weathering, including cell membrane biogenesis and cell cycle control. Moreover, some genes encoding flagellar, pilus, and polysaccharide biosynthesis were found (7). They help the bacterium to attach to the surface of the rock, which may play an important role in rock weathering. There are 410 genes with unknown functions, which may be related to rock weathering.

### Nucleotide sequence accession numbers.

This whole-genome shotgun project has been deposited at DDBJ/EMBL/GenBank under the accession no. JPFJ00000000. The version described in this paper is version JPFJ01000000.
